# LCGSC-YOLO: a lightweight apple leaf diseases detection method based on LCNet and GSConv module under YOLO framework

**DOI:** 10.3389/fpls.2024.1398277

**Published:** 2024-10-31

**Authors:** Jianlong Wang, Congcong Qin, Beibei Hou, Yuan Yuan, Yake Zhang, Wenfeng Feng

**Affiliations:** ^1^ School of Computer Science and Technology, Henan Polytechnic University, Jiaozuo, China; ^2^ School of Education, Henan Normal University, Xinxiang, China; ^3^ School of Computer and Information Engineering, Henan Normal University, Xinxiang, China

**Keywords:** apple leaf disease detection, coordinate attention, lightweight network, depth-wise separable convolution, YOLO

## Abstract

**Introduction:**

In response to the current mainstream deep learning detection methods with a large number of learned parameters and the complexity of apple leaf disease scenarios, the paper proposes a lightweight method and names it LCGSC-YOLO. This method is based on the LCNet(A Lightweight CPU Convolutional Neural Network) and GSConv(Group Shuffle Convolution) module modified YOLO(You Only Look Once) framework.

**Methods:**

Firstly, the lightweight LCNet is utilized to reconstruct the backbone network, with the purpose of reducing the number of parameters and computations of the model. Secondly, the GSConv module and the VOVGSCSP (Slim-neck by GSConv) module are introduced in the neck network, which makes it possible to minimize the number of model parameters and computations while guaranteeing the fusion capability among the different feature layers. Finally, coordinate attention is embedded in the tail of the backbone and after each VOVGSCSP module to improve the problem of detection accuracy degradation issue caused by model lightweighting.

**Results:**

The experimental results show the LCGSC-YOLO can achieve an excellent detection performance with mean average precision of 95.5% and detection speed of 53 frames per second (FPS) on the mixed datasets of Plant Pathology 2021 (FGVC8) and AppleLeaf9.

**Discussion:**

The number of parameters and Floating Point Operations (FLOPs) of the LCGSC-YOLO are much less thanother related comparative experimental algorithms.

## 1 Introduction

As one of the favorite fruits, apples are highly nutritious and widely cultivated around the world (Hyson, 2011). China has been the global leader in apple production (Hu et al., 2022). The apple cultivation industry has performed a vital task in promoting the agricultural economy of China. However, various diseases during the growth period of apples make the containment and management of apple leaf diseases extremely challenging (Dhaka et al., 2021). The prevention of apple leaf diseases is crucial for apple growth. Farmers need to minimize the incidence of leaf diseases through effective measures to ensure quality of apple production (Roy and Bhaduri, 2021). Therefore, timely detection of apple leaf diseases is essential for disease prevention and control. It not only ensures the quality of the fruits but also contributes to the improvement of agricultural yield.

Traditional apple leaf disease detection methods primarily depended on eye observation to identify disease categories. But, the approach has the problem of high labor intensity. The method of manual visual inspection no longer meets the needs of modern agriculture for efficiency and precision. Thus, it is essential to introduce more advanced technologies and methods to achieve greater efficiency in disease detection (Arsenovic et al., 2019). With the advent of the machine learning-based technology, it has been employed in various aspects of agriculture (Tian et al., 2020; Attri et al., 2023; Elbasi et al., 2023). For example, Rastogi et al. classified leaves based on artificial neural networks and then graded them according to the number of diseases on the leaves (Rastogi et al., 2015). Ahmed et al. used a decision tree approach to detect the three most common rice diseases, which are black sigatoka, bacterial leaf blight and brown spot (Ahmed et al., 2019). Harakannanavar et al. combined K-Nearest Neighbor and image processing techniques for detecting leaf diseases in tomato plants (Harakannanavar et al., 2022). However, these machine learning-based methods are usually made less practical in embedded devices given the large amount of computations in the data preprocessing and feature extraction phases (Sujatha et al., 2021).

In recent years, deep learning techniques have made great progress in leaf disease detection (Ngugi et al., 2021; Khan et al., 2022; Bhuiyan et al., 2023; Kaur et al., 2022; Li et al., 2022b). Specifically, Jiang et al. utilized convolutional neural network (CNN) to obtain features from rice leaf diseases. Then, support vector machine (SVM) is employed to perform classification and prediction of specific diseases (Jiang et al., 2020). Zeng et al. addressed the challenges posed by complex environments and relatively small disease areas in crop disease images using a selfattentive convolutional neural network (SACNN) (Zeng and Li, 2020). With the emergence of target detection models, such as Faster-RCNN (Ren et al., 2015) and YOLO series (Redmon et al., 2016; Redmon and Farhadi, 2017), they can accurately detect the category and location of the target, which attracts more and more researchers to employ it in agriculture for crop spots on leaves for accurate classification and localization. However, the majority of disease detection models have a large number of parameters that are not well suited for deployment on mobile devices, which makes them difficult to meet the practical requirements of agricultural applications (Maddikunta et al., 2021; Johannes et al., 2017). In addition, Jiang et al. designed the INAR-SSD module for detecting apple leaf diseases, and the detection capability of the SSD network on various leaf diseases was enhanced by designing the inception module (Jiang et al., 2019). Due to the stacking of a large number of inception modules, INAR-SSD is not suitable for mobile devices. Therefore, in the last three years, researchers were largely focused on reducing the complexity of models to enhance the practicality. For instance, Bi et al. adopted a lightweight method for apple leaf disease detection by employing the MobileNet model (Bi et al., 2022). However, the presence of numerous convolutions and bottleneck modules still causes a substantial number of parameters. Barman et al. introduced a smartphone-based model for classifying citrus leaf diseases (Barman et al., 2020). Although the model was deployed on mobile devices, its application was limited to indoor experimental data, restricting its use in the practical detection of leaf diseases in complex outdoor environments. Hu et al. employed a lightweight method based on knowledge distillation to detect maize leaf diseases, which decreased the complexity of the model. But, it is difficult to guarantee the applicability of this method in real-world environments, which contain changes in weather and light (Hu et al., 2023). Xu et al. devoted to reducing the number of parameters and computation through effective model design in order to improve the efficiency of apple leaf disease detection. The study used three different categories of diseases and conducted experiments in dense scenarios as well as leaf shade scenarios. These research efforts provide an important foundation for disease detection. But only relying on these three disease categories and limited scenario setups may not be sufficient to deal with more complex real-world application. Therefore, expanding the disease categories and adding more complex scenario types can help to improve the generalization ability of the model so that it can better adapt to the diverse challenges of practical applications (Xu and Wang, 2023).

In modern agricultural production, the use of mobile devices to detect apple leaf diseases has become an important trend. The application of lightweight models has significantly improved the efficiency and feasibility of this process. By running these optimized models on mobile devices, farmers and agricultural experts are able to quickly and accurately identify diseases on apple leaves and take timely interventions accordingly. This real-time detection and rapid response capability is critical for crop health management, helping to not only increase yields but also improve the overall quality of produce. In addition, the application of lightweight models significantly reduces the reliance on expensive hardware equipment, further lowering the cost of detection. The popularity of mobile devices, coupled with the efficiency of lightweight models, has made disease detection more accessible and economical, providing a convenient solution for agricultural production. Through these improvements, modern agriculture is better able to achieve precise management and intelligent operations, increasing overall production efficiency and product quality.

To sum up, scholars have introduced numerous effective methods in the field of object detection, leading to significant advancements in the detection of plant leaf diseases (Jackulin and Murugavalli, 2022; Orchi et al., 2021). In order to solve the problems of the current apple leaf disease detection, such as large number of parameters and calculations, lengthy inference time, and difficulty in real-time monitoring, the paper proposes a lightweight network model LCGSC-YOLO that takes both detection speed and accuracy into account. The main contributions are as follows:

- The LCNet is utilized to reconstruct the backbone network, which mainly consists of lightweight depth-wise separable convolutions. These convolutions effectively reduce the number of model parameters and computations.
- The GSConv and VOVGSCSP modules are used to replace the original Conv and C3 modules in the neck network, which reduces the number of model parameters and computations while guaranteeing the fusion capability among different feature layers.
- The combination of coordinate attention and LCNet embedded in the tail of backbone makes the network achieves better feature extraction performance. Moreover, the coordinate attention is embedded behind each VOVGSCSP module to enhance the feature fusion capability of the network, which eventually ameliorates the problem of accuracy degradation caused by model lightweighting.

The later sections are organized as follows: the second part describes and shows the detailed contents of the dataset and elaborates on the methodology proposed in the paper. The third part analyzes and discusses the experimental procedures and results of this paper. The fourth section draws the conclusion of this paper.

## 2 Materials and methods

### 2.1 Datasets

**Figure 1:**
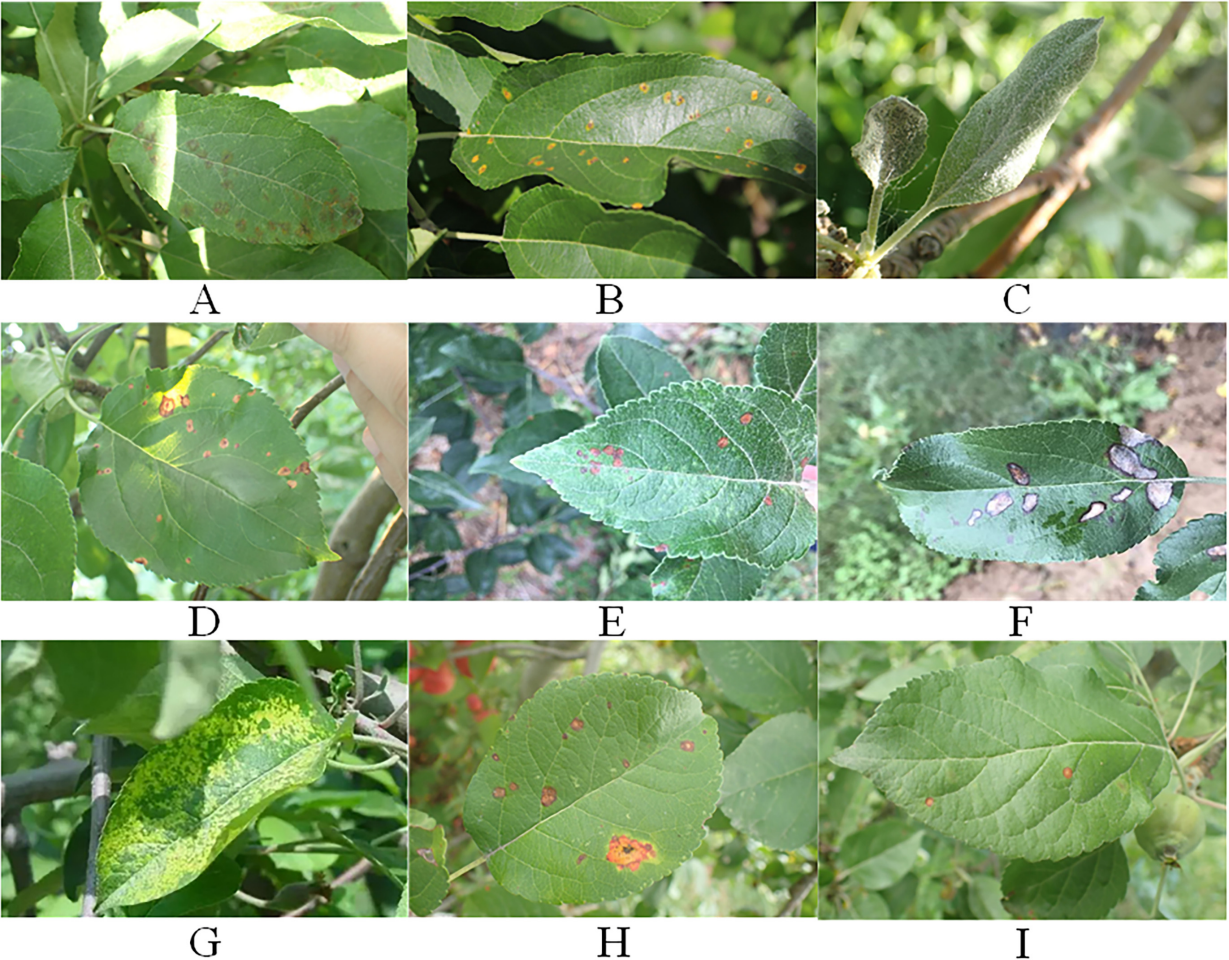
The representative images of different disease categories. **(A)** Scab. **(B)** Rust. **(C)** Powdery_mildew. **(D)** leaf spot. **(E)** Altermaria leaf spot. **(F)** Grey spot. **(G)** Mosaic. **(H)** Rust+ Frog_eye_leaf_spot. **(I)** Scab+ Frog_eye_leaf_spot.

The apple leaf disease data utilized in the paper have been selected from the Phytopathology 2021 (FGVC8) dataset and the AppleLeaf9 dataset. The images in the datasets are all derived from outdoor scenes. Seven common diseases have been chosen for the study. Frog_eye_leaf_spot, Powdery_mildew, Rust, and Scab were selected from FGVC8. Alternaria leaf spot, Grey spot, and Mosaic were selected from AppleLeaf9. Moreover, under natural conditions Frog_eye_leaf_spots are mixed with Rust and Scab to form two categories of disease occurrence scenarios, respectively. In total, there are seven disease names and nine disease categories. The specific number of images in each category is shown in **Table 1**. The representative images of different disease categories are shown in **Figure 1**.

**Table 1:** The number of images of different disease categories in the dataset.

Categories of leaf disease	Number		
	Original Images	Enhanced Images	Total
Frog_eye_leaf_spot	367	2569	2936
Powdery_mildew	400	2800	3200
Rust	313	2191	2504
Scab	460	3220	3680
Alternaria_leaf_spot	253	1771	2024
Grey_spot	163	1141	1304
Mosaic	145	1015	1160
Rust+Frog_eye_leaf_spot	107	749	856
Scab+Frog_eye_leaf_spot	82	574	656
Total	2290	16030	18320

All collected images were labeled using the LabelImg tool and saved in an XML file. In addition, the images in the datasets are enhanced with changes such as rotation, brightness, contrast, and addition of noise. Finally, there are 18320 images in the datasets, and the specific number of images in each category is shown in **Table 1**. During the datasets labeling process, this study has assumed all the browning areas on the leaves to be spots caused by pests and diseases. However, this study recognizes that browning may also be caused by other reasons such as environmental factors or human intervention, and this simplifying assumption may limit the comprehensiveness of the labeling and the generalization ability of the model. In order to improve the accuracy and rigor of the study, this study plans to introduce a detailed comparative analysis of types of browning not caused by pests and diseases in future studies. This will help understand the multiple causes of browning and make appropriate adjustments in datasets labeling to ensure that the model can accurately identify and distinguish among various browning types and improve overall detection. The links to the datasets used in this study are provided below: https://drive.google.com/drive/folders/1MRfK5eOm5-6KZTngPzpzjp9gx1NyEvZY?usp=sharing.

As illustrated in **Figure 1**, apple leaf disease detection faces several challenges. Firstly, different categories of leaf diseases have different shapes and sizes, which makes feature extraction more difficult. Secondly, most leaf diseases are small and densely distributed, which increases the difficulty of the localization process. Finally, under outdoor conditions, natural light and raindrops may interfere with leaf disease recognition.

A series of different scenes were selected as part of the experimental datasets for this study. The dark scene simulated the detection of leaf diseases in a low light or night environment. The rainy scene symbolized the effects of rain on the leaf surface, such as raindrop shading and water droplet retention. The lighting scene emphasizes the situation of leaves under direct sunlight or bright light. The dense scene contains a large number of diseases, which is closer to the leaf disease situation in real farms and provides a more rigorous testing environment for evaluating the performance of the detection algorithms. The multiple leaves scene and the two-spots scene further increase the complexity of the scenarios by taking into account multiple leaves and the interactions between two spots on the leaves. By conducting experiments in these different scenarios, the applicability of the proposed leaf disease detection method in practical applications can be comprehensively evaluated and more reliable technical support can be provided for agricultural production.

### 2.2 Design for LCGSC-YOLO

**Figure 2:**
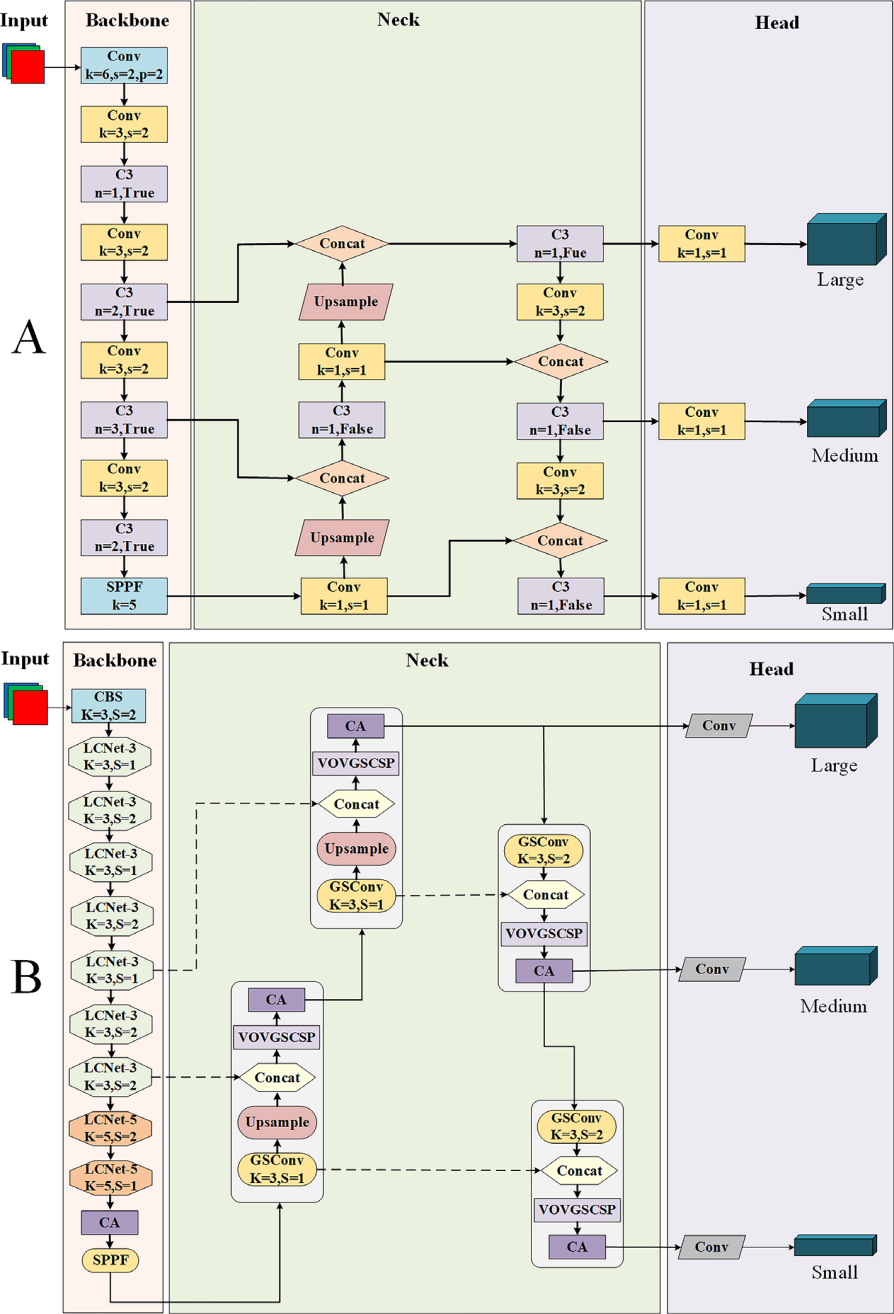
The framework of the two models. **(A)** The YOLO model. **(B)** The proposed LCGSC-YOLO model.

With the aim of achieving a lightweight model on apple leaf disease detection to make it more convenient to be applied to embedded devices, the paper proposes a lightweight method and names it LCGSC-YOLO. **Figures 2A, B** show the detailed framework of YOLOv5 and the proposed LCGSC-YOLO in the paper, respectively.

YOLO is chosen as the main framework in this study mainly because of its efficiency and real-time performance. YOLO is able to predict both bounding box and class probabilities of targets in a single network, which allows it to perform well when dealing with complex backgrounds and dense targets while maintaining low computational requirements and fast inference speed. Some other target detection framework, although advantageous in different aspects, such as SSD performs better in terms of speed but may not be as good as YOLO when dealing with small targets and complex backgrounds. Faster R-CNN, although it performs well in terms of detection accuracy, its two-phase structure results in slower inference and higher computational requirements, which limits its application in real-time detection. Taking these factors into consideration, YOLO is chosen in this study to meet our requirements for real-time performance and computational efficiency, while effectively handling complex backgrounds and high-density targets. The YOLOv5 version is chosen because it has demonstrated excellent stability and maturity in the field of target detection, providing a solid foundation for innovation in this study.

YOLOv5 is made up of four parts: the input layer, the backbone network, the neck network and the prediction head. As presented in **Figure 2A**, it can be seen that the backbone network of YOLOv5 stacks numerous Conv and C3 modules. The Spatial Pyramid Pooling-Fast (SPPF) module is utilized to capture multi-scale target information and then connects to the neck network (Zhang et al., 2023). In the neck network, besides the Conv and C3 modules, the Concat module is employed to aggregate the feature maps of different layers, thus reducing feature map information loss. The detection head module mainly performs multi-scale target detection of feature maps (Sun et al., 2022).

Due to the large number of Conv and C3 modules in the original YOLOv5 framework, it is difficult to embed it for utilizing in mobile devices (Xu and Wang, 2023). Therefore, an efficient LCGSC-YOLO for apple leaf disease detection is proposed in the work. The framework of LCGSC-YOLO is shown as **Figure 2B**. Compared to YOLOv5, the main innovations of LCGSC-YOLO are described below: 1) LCNet is used to reconstruct the backbone network and is categorized into LCNet-3 and LCNet-5 according to the convolutional kernel size. LCNet greatly reduces the number of parameters and computations of the model. 2) In the neck network, we are utilizing the GSConv module and the VOVGSCSP module to replace the Conv and C3 modules from the YOLO framework. 3) The coordinate attention(CA) is inserted at the tail of the backbone and after each VOVGSCSP module, which alleviates the problem of detection accuracy reduction caused by the lightweighting of the model.

In the next section, the effectiveness of each module will be analyzed step by step. Firstly, this study demonstrates that the lightweight design of LCNet effectively reduces the computational complexity through theory and formulas. Similarly, this study analyzes that GSConv optimizes the convolution operation by mixing convolution kernels, which further reduces the computation. Secondly, the introduction of CA attention mechanism makes the accuracy of the model improved. Finally, the experimental results show that the combination of LCNet and GSConv makes a significant reduction in the number of parameters and the number of Floating Point Operations (FLOPs) of the model, while the CA attention mechanism further enhances the performance and accuracy of the model.

#### 2.2.1 Design of the LCNet module

**Figure 3:**
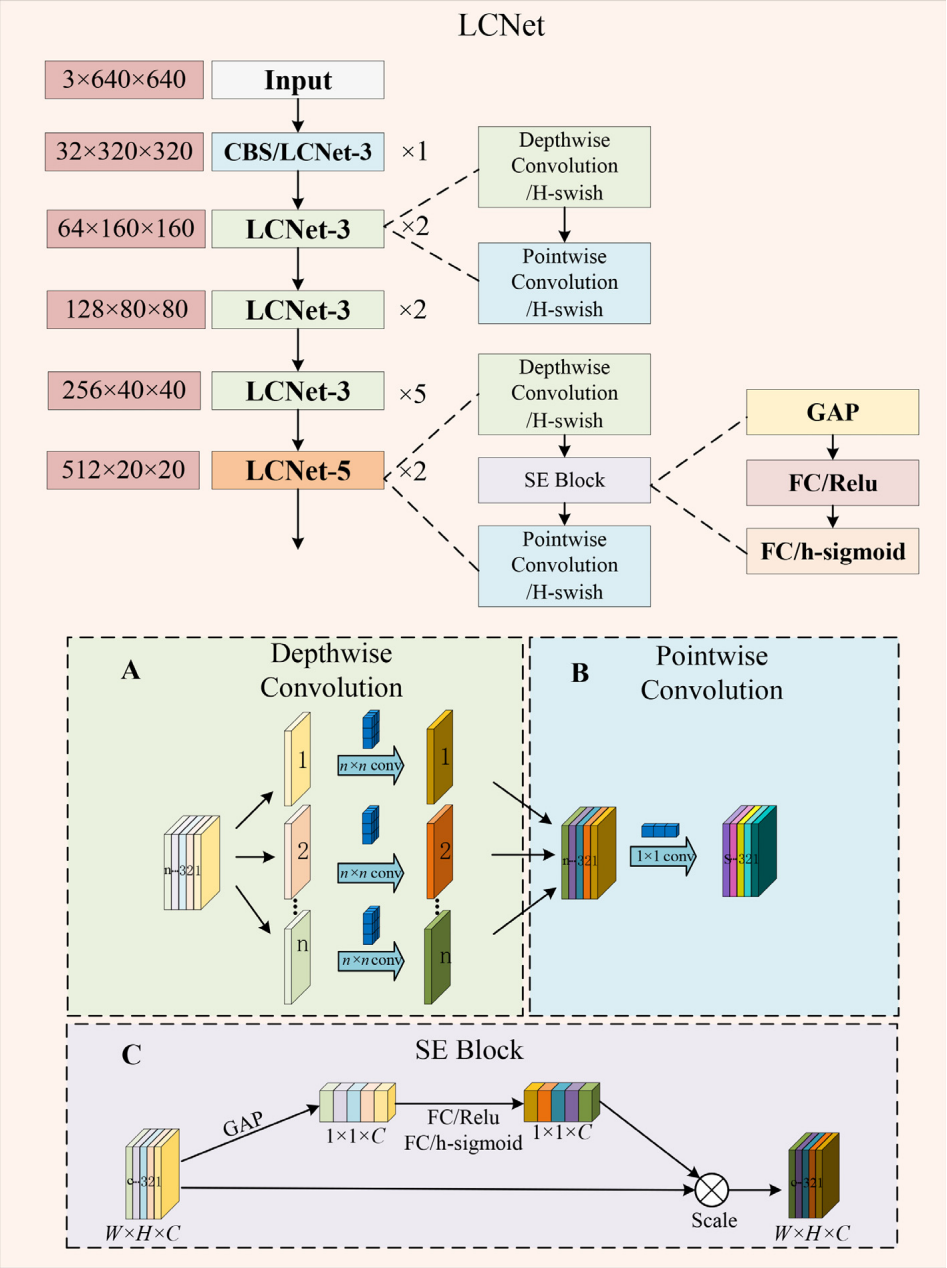
The structure of the LCNet module. **(A)** Depth-wise Convolution. **(B)** Pointwise Convolution. **(C)** SE Block.

To be more conveniently applied to embedded devices, the paper compresses the model parameters as much as possible with the guarantee of relatively high detection accuracy. The backbone of LCGSC-YOLO is structured by utilizing LCNet (Cui et al., 2021). The LCNet is utilized to decrease the number of parameters and computations in the feature extraction process. As shown in **Figure 3**, depending on the size of the convolutional kernel, the LCNet is divided into two types of modules: LCNet-3 and LCNet-5. The LCNet-3 has only 3 × 3 depth-wise (DW) convolution module and point-wise (PW) convolution module to extract features, while the LCNet-5 utilizes 5 × 5 convolution and introduces a squeeze-and-excitation (SE) attention module.

The 5 × 5 convolution used in the LCNet-5 module captures a larger range of features which helps to recognize more complex patterns in an image, especially when local features are not sufficient to describe the overall structure. Although a single 5 × 5 convolutional kernel has a large number of parameters, the total number of parameters may be relatively small compared to the use of multiple 3 × 3 convolutional kernels in the same target region. The introduction of the SE module enables the fusion of information between different channels and improves the accuracy of model detection.

Within each LCNet-3 and LCNet-5 module, the first layer of the network performs a down-sampling operation on the feature maps to reduce the size of the feature maps to one-half of their original size. In addition, the number of input feature map channels is expanded to twice the original number. The subsequent layers extract only the holdout features without modifying the width and height of the feature map as well as the number of channels. The working principle of LCNet-3 and LCNet-5 is shown in **Figure 3**.

Depth-wise separable convolution is divided into two components: the first is the depth-wise convolution and the other is the point-wise convolution (Chollet, 2017). Depth-wise convolution is a 2D convolution of each channel from the input image to reduce the number of parameters. Point-wise convolution uses 1 × 1 convolution for all channels based on depth-wise convolution, which greatly reduces the amount of computation. The schematic diagrams of depth-wise convolution and point-wise convolution are shown in **Figures 3A, B**, respectively.

In the following, computing the number of floating point operations (FLOPs) helps to illustrate and compare the complexity of standard convolution and depth-wise separable convolution.

Assume that the convolution kernel size is $A_{k}\times A_{k},A_{i}\times A_{i}\times C_{1}$ is the size of the input feature map, and the size of the output feature map is $A_{i}\times A_{i}\times C_{2}$ .

The number of standard convolution calculations is indicated as Equation 1:


(1) $$FLOPs\left(S\right)=A_{i}\times A_{i}\times C_{1}\times C_{2}\times A_{k}\times A_{k}$$

The number of depth-wise convolution calculations can be written as Equation 2:


(2) $$FLOPs\left(D\right)=A_{i}\times A_{i}\times C_{1}\times A_{k}\times A_{k}$$

Equation 3 demonstrates the number of pointwise convolution calculations:


(3) $$FLOPs\left(P\right)=A_{i}\times A_{i}\times C_{1}\times C_{2}$$

The number of depth-wise separable convolution calculations is displayed by Equation 4:


(4) $$FLOPs\left(DP\right)=A_{i}\times A_{i}\times C_{1}\times A_{k}\times A_{k}+A_{i}\times A_{i}\times C_{1}\times C_{2}$$

The ratio of the number of depth-wise separable convolution to the number of standard convolution calculations is:


(5) $$\frac{FLOPs\;(DP)}{FLOPs\;(S)}=\frac{A_{i}\times A_{i}\times C_{1}\times A_{k}\times A_{k}+A_{i}\times A_{i}\times C_{1}\times C_{2}}{A_{i}\times A_{i}\times C_{1}\times C_{2}\times A_{k}\times A_{k}}=\frac{1}{C_{2}}+\frac{1}{A_{k}\times A_{k}}$$

According to the Equation 5, the depth-wise separable convolutions can achieve a reduction in computations depending on the number of output channels and the size of the convolution kernel. Thus, depth-wise separable convolutions can greatly decrease the computations when the number of network layers is continuously increasing.

SE attention improves the performance of neural networks (Hu et al., 2018). As shown in **Figure 3C**, the SE attention involves two primary processes: squeeze and excitation. In the squeeze phase, global average pooling is first performed on the feature maps of each channel. It generates a single weight for each channel and the purpose of this step is to integrate the global information for each channel. In the excitation step, two fully connected layers are introduced. The output of these layers is passed through an activation function that produces a weight. Then, the weight is applied to the original feature map, which effectively assigns different importance to each channel.

Therefore, the LCNet-3 and LCNet-5 modules reconstruct the proposed lightweight backbone network of LCGSC-YOLO for fast feature extraction. Compared with the original YOLO framework, LCNet as the backbone network can dramatically decrease the number of parameters and the computations.

#### 2.2.2 Design of GSConv and VOVGSCSP modules

For a further reduction of the parameters and computations of the model, the GSConv module and the VOVGSCSP module are used to replace the original Conv and C3 modules, which are embedded in the neck network.

**Figure 4:**
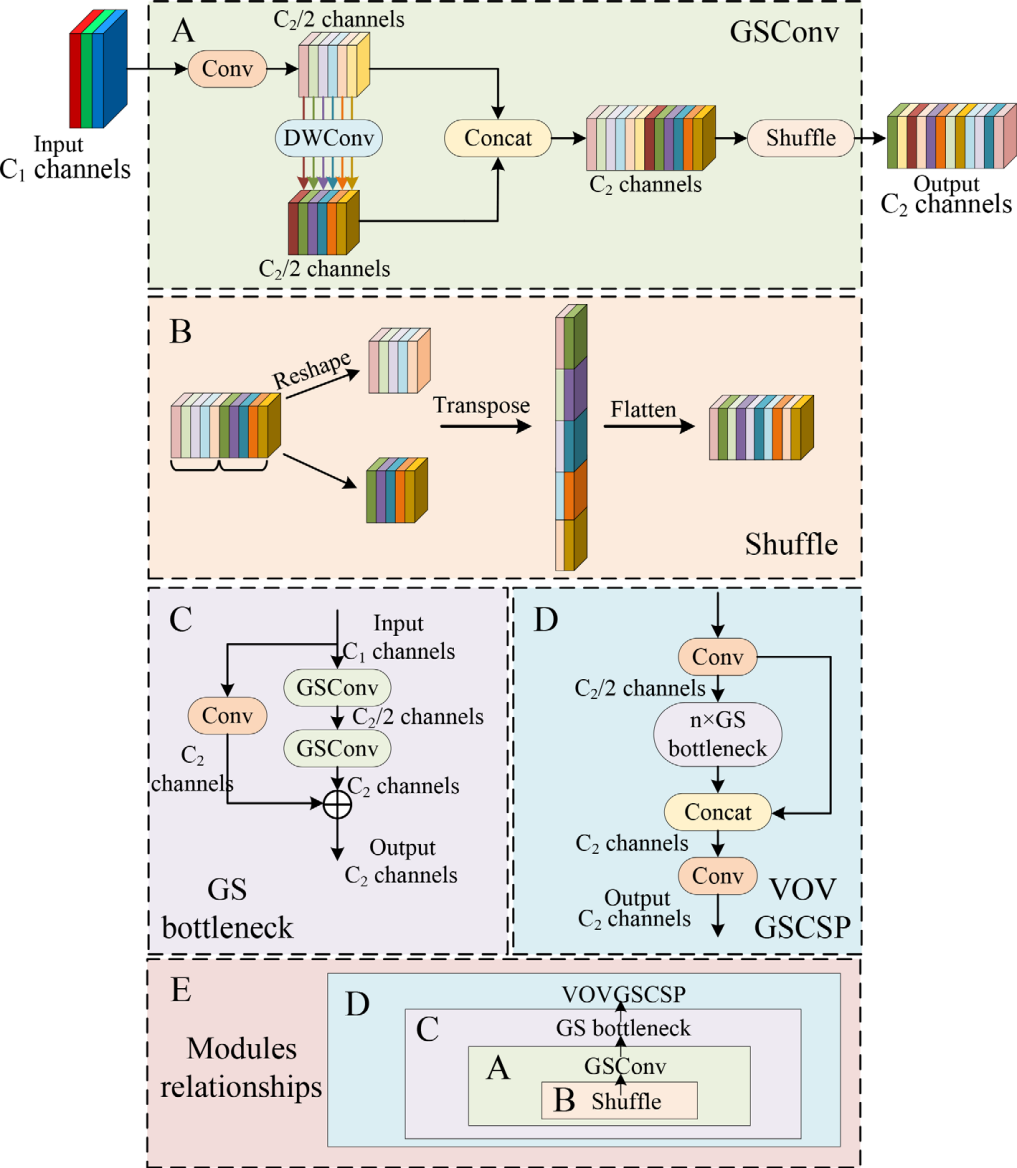
The structure of GSConv and VOVGSCSP modules. **(A)** GSConv. **(B)** The channel shuffle operation. **(C)** GS bottleneck. **(D)** VOVGSCSP. **(E)** Modules relationships.

GSConv is a convolution strategy in depth-wise separable convolution (Li et al., 2022a). It has less parameters and cheaper computation cost than standard convolution. (convolution + BN + activation function). The implementation flow of GSConv is shown in **Figure 4A**. In the basic module of GSConv, the number of input channels is $C_{1}$ and the number of output channels is $C_{2}$ . Firstly, the input is processed by standard convolution to change the number of channels to $C_{2}/2$ , which generates a hidden feature map with fewer channels and reduces the number of parameters. Then, the hidden layer is processed using DW convolution and the number of channels remains $C_{2}/2$ . Next, the result after the first standard convolution is connected with the result after DW convolution by Concat operation. Finally, the shuffle operation is introduced to achieve fast fusion of information among different channels, which enhances the extracted semantic information. The shuffle operation is shown in **Figure 4B**.

Here, we also provide a brief analysis of FLOPs. Suppose that the output feature map width and height are denoted as *W* and *H*, respectively. $K_{1}$ means standard convolutional kernel size and $K_{2}$ represents DW convolutional kernel size. $C_{1}$ and $C_{2}$ indicates the number of channels for feature map input and output, respectively.

Equation 6 represents the number of standard convolution calculations:


(6) $$FLOPs\left(SC\right)=W\times H\times K_{1}\times K_{2}\times C_{1}\times C_{2}$$

The number of GSConv calculations is demonstrated in Equation 7:


(7) $$FLOPs\left(GS\right)=W\times H\times K_{1}\times K_{2}\times \left(C_{1}+1\right)\times C_{2}/2$$

Ratio of GSConv to standard convolution calculations:


(8) $$\frac{FLOPs\left(GS\right)}{FLOPs\left(SC\right)}=\frac{W\times H\times K_{1}\times K_{2}\times \left(C_{1}+1\right)\times C_{2}/2}{W\times H\times K_{1}\times K_{2}\times C_{1}\times C_{2}}=\frac{C_{1}+1}{2C_{1}}$$

From Equation 8, it is possible to draw the following conclusions. As the number of channels continues to increase, the FLOPs of GSConv are nearly half that of standard convolution. Due to the increase in the number of input image channels in the LCGSC-YOLO model after backbone feature extraction, the number of feature map channels is raised from 3 to 512, as shown in **Figure 3**. When the number of channels is 512, the computations amount of GSConv is almost close to half that of the standard convolution. Therefore, the application of the GSConv module will reduce the computations significantly over the standard convolution.

Next, GSConv is utilized to form the GS bottleneck, which shown as in **Figure 4C**. It consists of two GSConv layers. The first GSConv layer halves the number of channels. Further, the output is residually concatenated with the former GSConv. Finally, the VOVGSCSP module consists of multiple GS bottlenecks modules. In the VOVGSCSP module, the Conv module compresses the channel number to one-half of the original number. Then, the result after the GS bottlenecks module is concatenated with the result after the Conv module. The network diagram of the VOVGSCSP is displayed in **Figure 4D**.

As a result, we choose to merge GSConv into the Neck network with a large number of channels. Specifically, the GSConv module and the VOVGSCSP module are utilized to substitute the original Conv and C3 modules, which can significantly reduce the computations. In addition, **Figure 4E** shows the relationship of the connections among the modules.

#### 2.2.3 The introduction of coordinate attention module

By improving the lightweighting of each module of the YOLO framework, the number of parameters and computations of the model can be dramatically decreased and the inference speed of the model can be improved. However, this inevitably brings about a degradation in model detection accuracy caused by the lightweighting of the model.

As a consequence, introducing a coordinate attention (CA) module (Niu et al., 2021) at key positions of the network is an effective strategy to increase the accuracy of the model to leaf disease. With the above operational improvements, the ability of the network to recognize and localize leaf diseases can be improved without adding too much computation.

**Figure 5:**
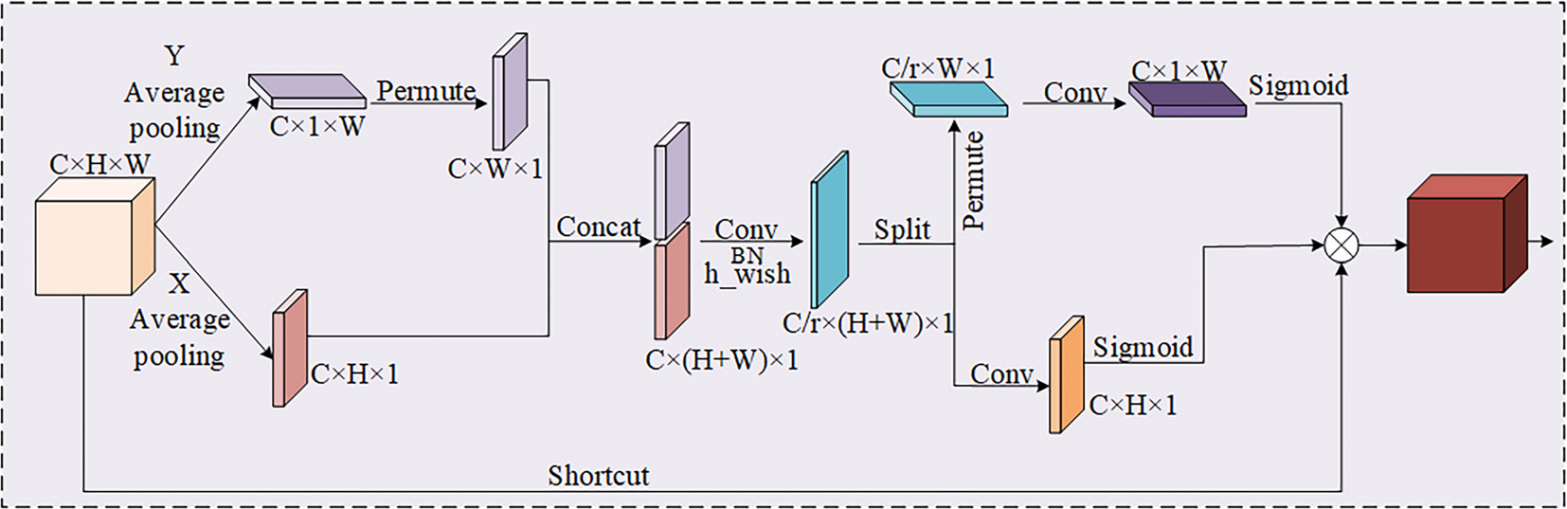
The structure of coordinate attention.

As shown in **Figure 5**, the CA attention mechanism performs feature extraction in both directions of the input feature map, which not only obtains the relationship among the channels, but also takes into account the positional information about the directions. It helps the model to better localize and identify the target. The feature information in both directions can be fused by Concat, and then non-linear activation is performed using the h_wish function to obtain intermediate features of the coded information. The intermediate information feature map is divided in both height and width directions to get two different dimension vectors. Finally, nonlinear activation is performed with a sigmoid function to generate the corresponding attentional weights.

In consequence, the coordinate attention (CA) is embedded in the tail of the backbone and the end of each VOVGSCSP module, which enhances the recognition capability of the model for various apple leaf diseases. CA attention is not only excellent in performance, but also has lightweight characteristics, which can be flexibly adopted in the corresponding network framework. As a result, it alleviates the accuracy loss from model compression without incurring substantial computational costs.

## 3 Experiments analysis and discussion

### 3.1 Implementations and settings

The experiments are performed in Ubuntu system with an Intel Xeon(R) Silver 4214R CPU@2.40 GHz x48 processor, 128 GB of RAM, a graphics card NVIDIA Corporation TU102GL [Quadro RTX 8000], CUDA 12.2, Pytorch 2.1.0, Python 3.9.18. The hyperparameters of the experiments are set as follows: the Epochs of the model are set to 300, the Initial learning rate of the model is given as 0.01, the model optimizer is selected as SGD, and the Batch size is fixed to 32. All apple leaf disease data are classified by a ratio of 7 to 2 to 1 as training set, validation set, and test set.

### 3.2 Evaluation indicators

Aiming to objectively evaluate the validity of the experimental results, the paper chooses mean accuracy precision (mAP), precision (P), recall (R) as the objective evaluation metrics of the experiment (Hossin and Sulaiman, 2015). The mAP denotes the sum of the mean accuracies of all categories divided by all categories. The P means the ratio of the actual number of positive samples in the predicted sample to the number of all positive samples. The R represents the ratio of the number of actual positive samples in the predicted sample to all predicted samples. The assessment metrics were calculated according to the following Equations 9–12.


(9) $$P=\frac{TP}{TP+FP}$$

(10) $$R=\frac{TP}{TP+FN}$$

(11) $$AP=\int_{0}^{1}P\left(R\right)dR$$

(12) $$mAP=\frac{\sum_{i=1}^{n}AP_{i}}{n}$$

TP (True Positive) indicate the number of positive samples that the model correctly predicts as positive. FP (False Positive) expresses the number of negative samples that the model incorrectly predicts as positive. FN (False Negative) signifies the number of positive samples that the model incorrectly predicts as negative (Zhu et al., 2023).

Moreover, the number of parameters (Para) and FLOPs are employed to assess the complexity of the model (Justus et al., 2018). Assume that the convolution kernel size is $A_{k}\times A_{k},A_{i}\times A_{i}\times C_{1}$ is the size of the input feature map, and the size of the output feature map is $A_{i}\times A_{i}\times C_{2}$ . The fewer the number of parameters and FLOPs, and the lower the complexity of the model, which means it is more suitable to be applied in resource-constrained embedded devices.

The number of depth-wise separable convolution parameters is indicated as Equation 13:


(13) $$Para\left(DP\right)=A_{k}\times A_{k}\times C_{1}+C_{1}\times C_{2}$$

The calculation formula of FLOPs for depth-wise separable convolution is shown in Equation 4.

Finally, frames per second (FPS) is utilized to evaluate the inference speed of the model (Kiani Galoogahi et al., 2017), larger values of FPS indicate that more data is processed within the same time.

### 3.3 Ablation experiments

A series of testing experiments validate the effectiveness of the different lightweighting modules proposed in the paper. In order to ensure the generalization ability of the model, this study has adopted the method of dividing the validation set. The datasets have been divided according to 70% for training, 20% for validation, and 10% for testing. With this validation strategy, this study evaluated the performance of the model on unseen data. Test 1 indicates YOLOv5s, which is treated as a baseline model. Test 2 means reconstructing the backbone of the YOLO by LCNet. Test 3 demonstrates the adoption of the GSConv module and the VOVGSCSP module to replace the Conv and C3 modules in the YOLO framework, respectively. Test 4 indicates a combination of improvements from Test 2 and Test 3. The results of the objective assessment of the model detection performance with different improvement modules are listed in **Table 2**.

**Table 2:** The results of ablation experiments with different lightweighting improvement methods.

	Model	P/%	R/%	mAP/%	Para/M	FLOPs/G	FPS
Test 1	YOLOv5s	**93.8**	**93.4**	**96.6**	7.03	16.0	35
Test 2	YOLO+LCNet	92.9	92.2	95.3	3.64	6.3	56
Test 3	YOLO+GS_VOV	93.6	92.5	96.1	6.04	14.2	39
Test 4	YOLO+LCNet+GS_VOV	92.1	91.6	94.2	**2.61**	**5.6**	**60**

Bold values represents the best performance.

The conclusion can be obtained by analyzing the experimental results of Test 1 and Test 2. In comparison for YOLOv5s, the number of parameters and FLOPs of YOLO-LCNet are decreased by 48.22% and 60.63%, respectively, and increases the FPS by 60%. It demonstrates that utilizing the LCNet module to reconstruct the backbone network of the YOLO framework can dramatically decrease the complexity of the model and can quite obviously increase the inference speed of the model. From Test 1 and Test 3, the modified neck network of YOLO with GSConv_VOVGSCSP module (YOLO+GS_VOV) reduces the number of parameters and FLOPs by 0.99M and 1.8G, respectively, while mAP is reduced by only 0.5%. The consequences confirm that the introduction of the proposed GSConv_VOVGSCSP (GS_VOV) module into the neck network not only makes the model more lightweight but also has little effect on the feature fusion capability among the different network layers in the neck network. Based on the experimental results of Test 4, it can be observed that the number of model parameters and FLOPs in the YOLO framework introduced by LCNet together with the GS_VOV module are only 37.12% and 35% of those in YOLOv5s, respectively. The model inference speed attained 60 FPS, which is 1.71 times faster than that of YOLOv5s. The above discussion and analysis demonstrate the effectiveness of the proposed method.

It is clear from **Table 2** that the lightweighting of the model inevitably brings about the problem of detection accuracy degradation. Therefore, it is very necessary to improve the detection accuracy as much as possible without bringing in higher computations.

### 3.4 Discussion of different attention mechanisms

We take YOLO+LCNet+GS_VOV as the baseline network and verify the influence in model performance after introducing different attention modules. Test 4 is YOLO+LCNet+GS_VOV, which combines the LCNet, GSConv and VOVGSCSP modules. Test 5 represents the SE module that is added to Test 4, Test 6 indicates the CBAM module that is introduced to Test 4, and Test 7 is the proposed LCGSC-YOLO, which integrates the CA module from Test 4. The objective evaluation results of introducing different attention modules on the performance of YOLO+LCNet+GS_VOV are reflected in **Table 3**, respectively.

**Table 3:** Results of introducing different improvements in YOLO+LCNet+GS_VOV.

	Model	P/%	R/%	mAP/%	Para/M	FLOPs/G	FPS
Test 4	–	92.1	91.6	94.2	**2.61**	**5.6**	**60**
Test 5	+SE	92.8	91.7	94.7	2.81	5.9	58
Test 6	+CBAM	93.2	91.9	95.0	2.84	6.9	50
Test 7	+CA	**93.4**	**92.0**	**95.5**	2.96	6.7	53

Bold values represents the best performance.

Compared to Test 4, the results of Test 5, Test 6, and Test 7 demonstrate that adding the attention mechanism to YOLO+LCNet+GS_VOV can enhance the detection performance of the model. Although all the listed attention mechanisms improve detection accuracy, they simultaneously increasing the number of parameters and decreasing the model inference speed. However, the task at this stage is to maximize accuracy within a limited range of parameters variation. The key is to reach a balance between lightweighting and accuracy. Therefore, in this paper, CA is chosen at this stage because of the desire to increase the detection accuracy as much as possible. As can be observed in Test 4 and Test 7, the mAP of LCGSC-YOLO is 1.3% higher than that of YOLO+LCNet+GS_VOV, while the number of parameters and computations only increase by 0.35 M and 1.1 G, respectively. Even though there is some computational cost associated with the approach, it has very little effect on the inference speed of the model. LCGSC-YOLO inference speed is reduced by only 7 FPS. With the above results, the CA attention mechanism can effectively alleviate the problem of detection accuracy degradation caused by the model lightweighting. Therefore, the addition of CA attention mechanism to the proposed lightweight model can make the model performance more excellent.

After introducing the CA attention module in the lightweight model, the research further conducts ablation experiments by combining CA with above modules of lightweight to verify the independence of the CA module and to prove that there is no dependency among the modules. The results in **Table 4** show that Test 8 represents YOLO+GS_VOV+CA, Test 9 denotes YOLO+LCNet+CA, and Test 10 indicates YOLOv5s+CA. Compared with Test 3 in **Table 2**, Test 8 shows the increase in accuracy and recall after the introduction of the CA module, proving that the CA module plays an active role in model performance optimization. Similarly, the comparison between Test 9 and Test 2 shows that the introduction of the CA module improves the detection performance, which mAP improves to 95.9%. Test 10 further demonstrates the effectiveness of the CA module in the YOLOv5s model, with mAP reaching 97.1%. The above experimental results validate that the CA module is able to produce improvements on multiple lightweight modules. However, although the detection performance is improved, this improvement is accompanied by both increase in the number of parameters and computational complexity. Therefore, there is a balance between accuracy and model complexity.

**Table 4:** Results of ablation experiments after introduction of CA attention.

	Model	P/%	R/%	mAP/%	Para/M	FLOPs/G	FPS
Test 7	–	93.4	92.0	95.5	**2.96**	**6.7**	**53**
Test 8	YOLO+GS_VOV+CA	93.9	92.9	96.5	6.38	15.1	37
Test 9	YOLO+LCNet+CA	93.2	92.8	95.9	4.18	6.9	52
Test 10	YOLOv5s+CA	**94.5**	**93.8**	**97.1**	7.26	16.8	33

Bold values represents the best performance.

As a conclusion, LCGSC-YOLO has several advantages. Firstly, the model with small number of parameters do not require a large amount of storage space. Secondly, the model is low computation and can be run with limited hardware resources. Finally, the model training and inference speed is fast and can process the data quickly. Therefore, LCGSC-YOLO is more suitable to be deployed in embedded devices for detecting apple leaf diseases.

### 3.5 The selection of lightweight backbone networks

In this subsection, aiming to validating the performance of different backbone networks, the backbone of the YOLO framework is reconstructed by the current mainstream lightweight modules. YOLO-MN3 means the backbone of YOLO is constructed by employing the MobileNetv3 module (Howard et al., 2019), and YOLO-SN2 illustrates that it is reconstructed by applying the basic modules of ShuffleNetv2 (Ma et al., 2018), YOLO-GN and YOLO-EN2 denote that the YOLO backbone is composed of modules applying GhostNet (Khan et al., 2022) and EfficientNetv2 (Tian et al., 2020), respectively, and the backbone with the LCNet is known as YOLO-LCNet. **Table 5** lists the results of the test experiments for different lightweight backbone networks.

**Table 5:** Comparison of experimental results of different lightweight improved backbone.

Model	P/%	R/%	mAP/%	Para/M	FLOPs/G	FPS
YOLOv5s	**93.8**	**93.4**	**96.6**	7.03	16.0	35
YOLO-MN3	92.2	91.5	95.3	3.91	7.2	48
YOLO-SN2	93.2	91.6	95.5	3.85	6.9	48
YOLO-GN	93.5	93.3	96.2	5.39	8.6	43
YOLO-EN2	93.3	92.7	95.7	3.78	6.5	54
YOLO-LCNet	92.9	92.2	95.3	**3.64**	**6.3**	**56**

Bold values represents the best performance.

As can be noticed from **Table 5**, all of listed different lightweight modules can to some degree decrease the number of parameters and computations of the model, but inevitably causes a loss of accuracy. Compared with YOLO-MN3, YOLO-SN2, YOLO-GN and YOLO-EN2, YOLO-LCNet has lower model complexity and quicker inference speed. Specifically, the mAP of YOLOv5 is only 1.3% higher compared to YOLO-LCNet, but the number of parameters and FLOPs of YOLO-LCNet are 48.22% and 60.63% lower than that of YOLOv5, respectively. In addition, compared to YOLOv5, the model inference speed of YOLO-LCNet is 60% faster than it. In a word, Using LCNet to reconstruct the backbone network of YOLO is a better choice to reduce model complexity and enhance model inference speed.

### 3.6 Comparative experiments

In this section, the experimental results of the proposed method are compared with other methods related to leaf disease detection. Specifically, INAR-SSD (Jiang et al., 2019) as a detection model for apple leaf disease detection with the ALDD dataset. BTC-YOLOv5s (Li et al., 2023) and MGA-YOLO (Wang et al., 2022) are improved lightweight apple leaf disease detection models based on the FGVC8 datasets. Khan et al. employ YOLOv4 to apple leaf disease detection (Khan et al., 2022). The experimental results of different models are presented in **Table 6**. The reason for selecting the above comparison methods is that they have some similarities with the method proposed in this study in terms of theoretical and technical characteristics. For example, both INAR-SSD and YOLOv4 belong to the classical methods in the field of target detection.BTC-YOLOv5s and MGA-YOLO are improved versions based on a lightweight target detection model, and their design ideas and technical features have some similarities with the method proposed in this study. In addition, these comparative methods use similar datasets when dealing with leaf disease detection tasks, and all attempt to address common challenges in leaf disease detection, such as light variations and leaf disease morphological diversity. Therefore, by comparing with these methods, the innovations and usefulness of the method proposed in this study can be better assessed in the context of current research hot spots and technological trends, as well as its strengths and limitations in solving the leaf disease detection problem.

**Table 6:** Comparison of experimental results of different lightweighting methods.

Method	INAR-SSD	BTC-YOLOv5s	YOLOv4	MGA-YOLO	LCGSC-YOLO
Scab	85.9	91.2	91.7	94.5	**96.3**
Rust	82.3	89.1	90.2	92.8	**94.2**
Mosaic	83.5	88.7	89.8	93.1	**94.9**
Grey_spot	80.4	87.3	88.7	91.8	**92.9**
Powdery_mildew	92.1	93.4	94.5	96.8	**99.2**
Frog_eye_leaf_spot	90.4	92.6	93.1	95.9	**98.6**
Alternaria_leaf_spot	81.2	88.4	89.6	92.1	**92.7**
P/%	84.7	89.6	90.7	91.8	**93.4**
R/%	83.5	88.7	89.8	90.9	**92.0**
mAP/%	85.1	90.1	91.0	93.8	**95.5**
Para/M	23.62	15.8	60.81	11.26	**2.96**
FLOPs/G	89.62	53.16	44.8	28.4	**6.7**
FPS	7	12	14	21	**53**

Bold values represents the best performance.

As shown in **Table 6**, the proposed LCGSC-YOLO model has fewer number of parameters and computations compared with other experimental models. Moreover, it also shows excellent performance in terms of detection accuracy and inference speed. In comparison with INAR-SSD, LCGSC-YOLO achieves a 10.4% increase in detection accuracy and a 46 FPS improvement in inference speed. In addition, the proposed method has only 12.53% of the number of parameters and 7.47% of the FLOPs of INAR-SSD, respectively. Meanwhile, the comparison results with BTC-YOLOv5s and MGA-YOLO show that the detection accuracies of BTC-YOLOv5s and MGA-YOLO are 5.4% and 1.7% less than that of LCGSC-YOLO, respectively. Besides, the model inference speed of LCGSC-YOLO are 4.41 and 2.52 times faster than that of them, respectively. However, the parameter amount and FLOPs of LCGSC-YOLO are only 18.73% and 12.60% of those of BTC-YOLOv5s, and about a quarter of those of MGA-YOLO. In comparison of YOLOv4, LCGSC-YOLO has 57.85M and 38.1G fewer parameters and FLOPs, respectively, while the model inference speed of LCGSC-YOLO is 39 FPS more than YOLOv4. The above analysis results illustrate that the proposed method is superior to the comparative experimental methods in terms of comprehensive performance.

**Figure 6:**
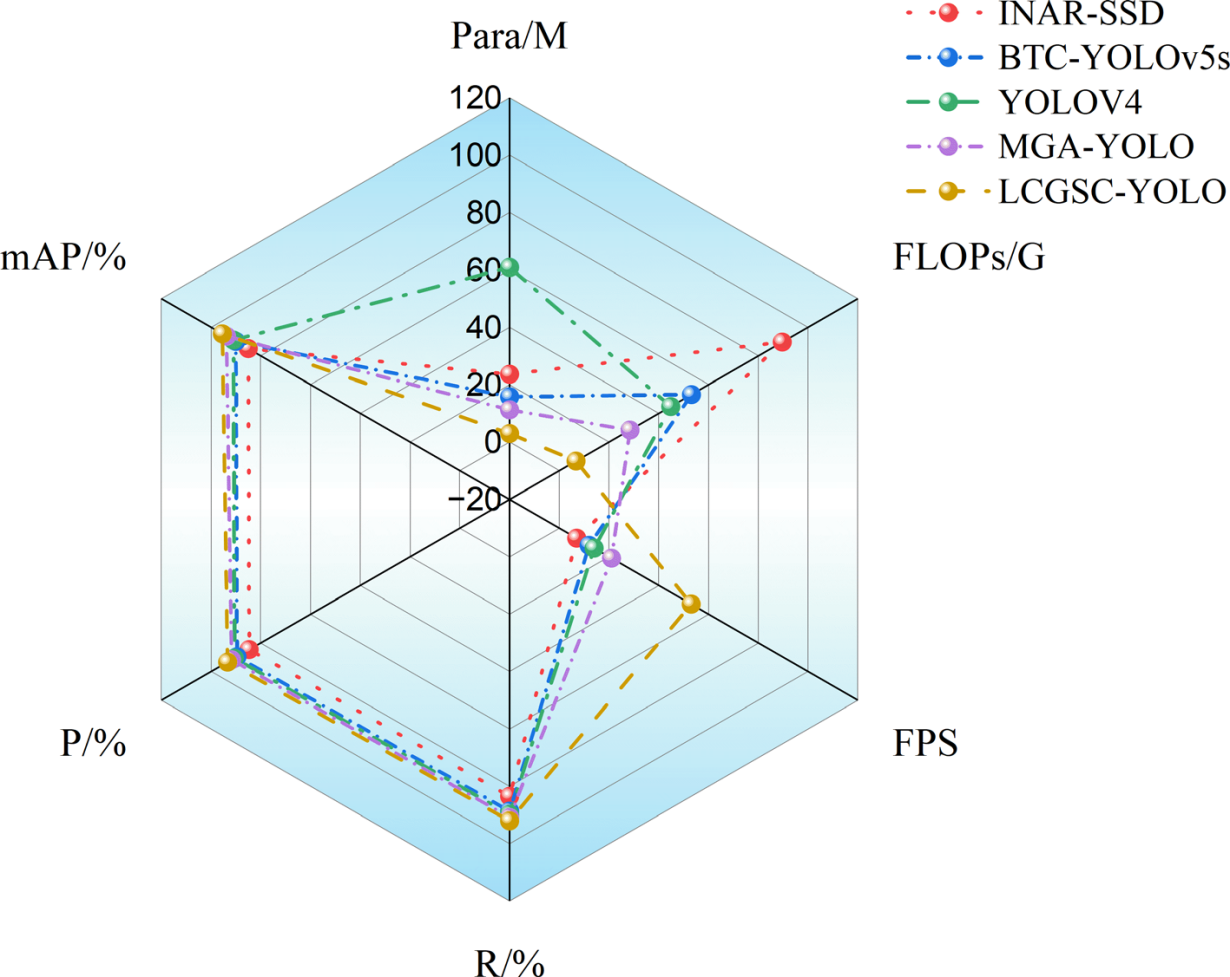
Radar plots showing the results of the five model tests.

**Figure 6** shows the radar charts of the different model experimental results. it can be concluded that INAR-SSD has the largest FLOPs, YOLOv4 has the largest number of parameters, and the mean average precision, precisions, and recalls of the different modelling methods are almost overlapping. In addition, The FPS of LCGSC-YOLO is obviously superior to the other methods, while the number of parameters and FLOPs are lower than those of the comparative experimental methods. In terms of the area surrounded by the test results of different models, LCGSC-YOLO mainly occupies the left area of the figure, which implies that LCGSC-YOLO has better detection performance while having fewer number of parameters and FLOPs.

**Figure 7:**
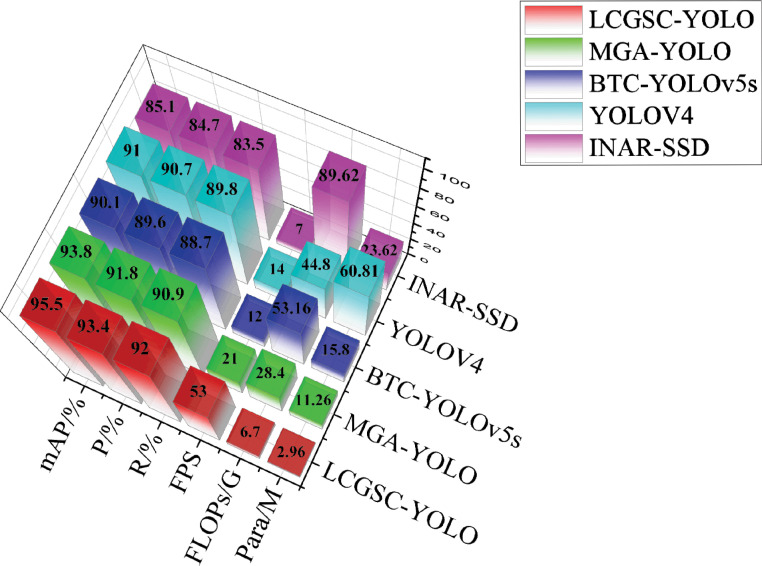
3D bar graphs of test results for five different models.

As illustrated in **Figure 7**, a 3D bar chart is drawn based on the test results of different models, which can visualize the comparison results of different models. It comes to a conclusion that the test results of different models do not show much difference in terms of mean average precision, precision, and recall. However, it can be observed that from INAR-SSD to LCGSC-YOLO are gradually increasing and decreasing in terms of the performance of FPS and FLOPs, respectively. It proclaims that the computational amount of the LCGSC-YOLO is gradually decreasing, and the inference speed is continuously increasing. Overall, compared with other models, LCGSC-YOLO has the lowest number of parameters and computational amount as well as the fastest inference speed.

**Figure 8:**
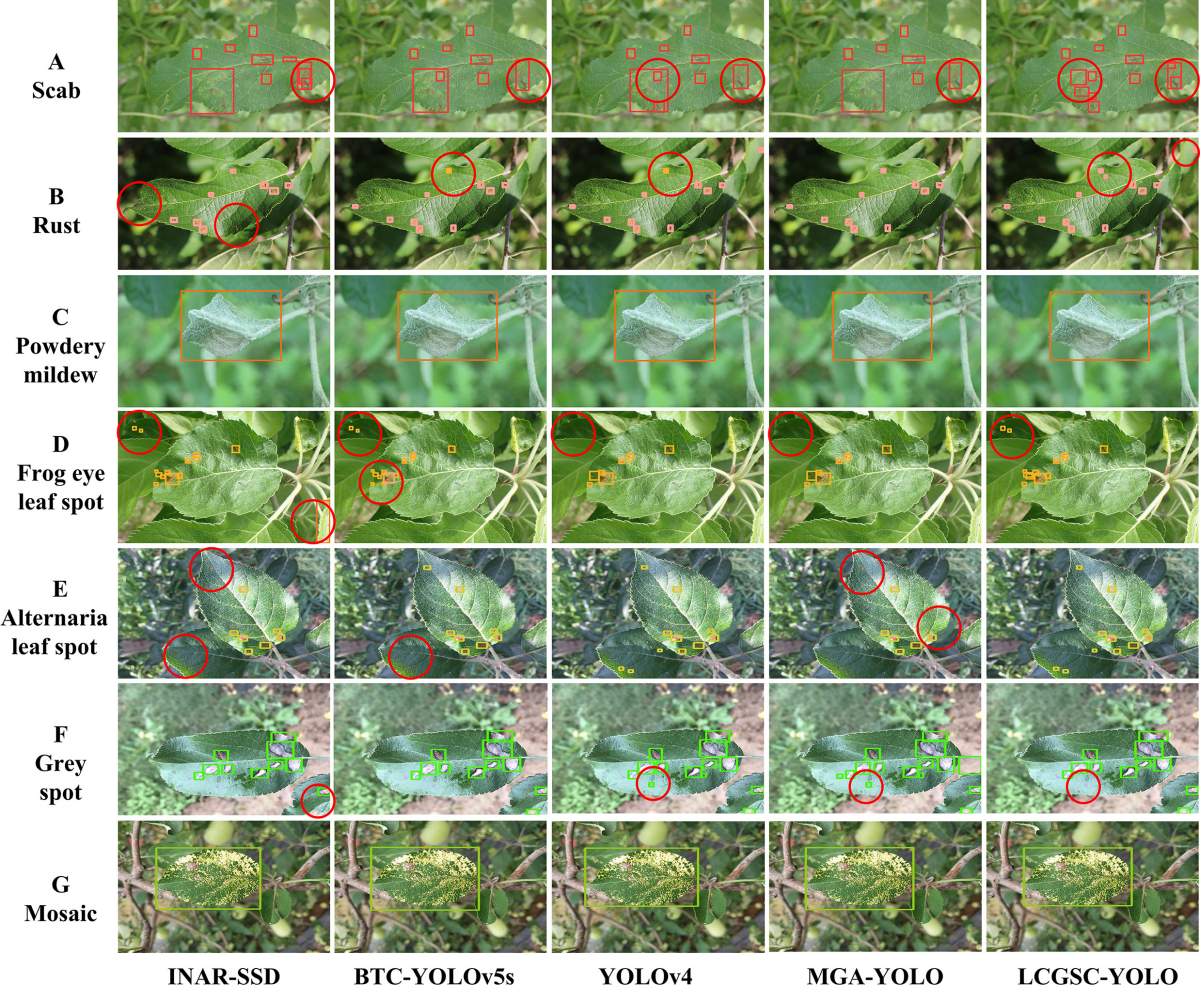
Comparison of different models for detecting apple leaf disease images. **(A-G)** indicates the name of the different diseases. The red circles show the contrasting positions.

To visualize the detection performance of the LCGSC-YOLO, **Figure 8** shows the detection results of INAR-SSD, BTC-YOLOv5s, YOLOv4 and MGA-YOLO and LCGSC-YOLO on different disease images, respectively. To improve readability, the detection information in **Figure 8** substitutes specific letters for disease names. A represents Scab, B represents Rust, C denotes Powdery_mildew, D denotes Frog_eye_leaf_spot, E indicates Alternaria leaf spot, and F indicates Grey spot, G means Mosaic. As shown in **Figure 8**, from the overall detection results, the proposed LCGSC-YOLO outperforms other algorithms in detecting different kinds of diseases. In particular, LCGSC-YOLO has better detection performance in detecting small diseases. To be specific, as indicated by the red circle from the Scab detection result images, it can be seen that LCGSCYOLO can separately detect adjacent disease regions, while other algorithms recognize them as a single disease. Moreover, it is apparent from the result images of Rust diseases that LCGSCYOLO can detect small disease areas at the edge positions. In addition, for the three diseases Frog_eye_leaf_spot, Alternaria leaf spot, and Grey spot, all methods have varying degrees of miss detection. In these three disease categories, it was difficult for all methods to identify all diseases due to the simultaneous presence of diseases in multiple leaves in the same scene. For Powdery_mildew and Mosaic, the detection results of all methods were almost the same.

**Figure 9:**
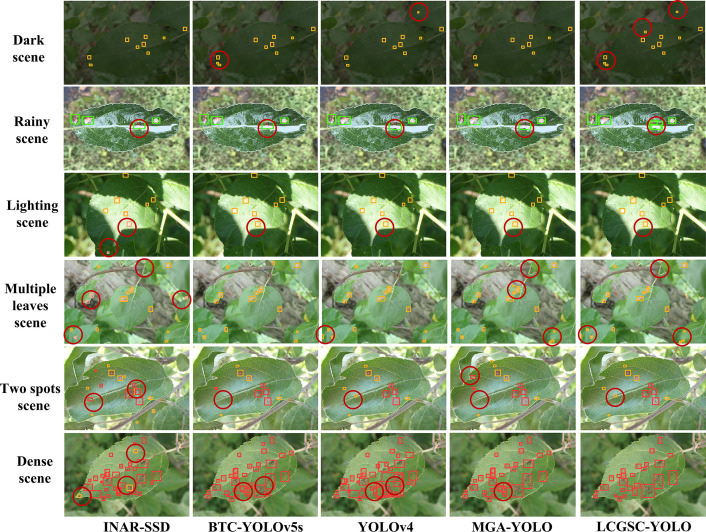
Comparison of the detection effects of different models on apple leaf disease images in special scenes. The different scenarios are represented from top to bottom.

To further illustrate the superiority of LCGSC-YOLO, **Figure 9** compares the model detection capabilities of INAR-SSD, BTC-YOLOv5s, YOLOv4, MGA-YOLO, and LCGSC-YOLO on specific scenarios. The scenes from top to bottom are dark, rainy, strong lighting, multiple leaves, two spots and dense scenes. As displayed in **Figure 9**, for disease images in dark scenes, the other four algorithms did not recognize small diseases, while LCGSC-YOLO was able to recognize them. For images of rainy scenes, LCGSC-YOLO accurately detected Grey spot that were difficult to recognize due to rainfall reflection, but the other four algorithms identified the disease spot as the same as adjacent disease spots. In the detection results of two spots scenes and multiple leaves scenes, there were varying degrees of missed detections. Due to the small size and dispersion of all diseases, it is difficult for all methods to identify all diseases. In lighting scenes, INAR-SSD incorrectly identifies a disease in the light. In addition, in dense scenes, LCGSC-YOLO can identify adjacent diseases separately. From the aforementioned analysis, it can be concluded that LCGSC-YOLO also has comparably equally excellent detection performance in special scenarios.

In general, by combining the detection capabilities of different models in different scenarios in **Figures 8**, **9**, it is obvious that LCGSC-YOLO shows excellent performance in detecting apple leaf diseases. The model utilizes fewer parameters and lower computational effort to achieve efficient leaf disease detection, and especially excels in model lightweighting. However, despite the obvious advantages of LCGSC-YOLO in terms of lightweighting, it is still important to note that its performance may be degraded when dealing with complex scenarios such as dense diseases, multiple leaves or two spots. In addition, the model showed missed detection in terms of detecting tiny diseases, which requires further optimization of the method. Therefore, in the future, the study will continue to explore the optimization of the LCGSC-YOLO model to improve its robustness and adaptability, and to further optimize its detection accuracy for more comprehensive and stable apple leaf disease detection.

## 4 Conclusions

To address the issues of complex background and high model complexity of apple leaf disease detection in natural scenes, the paper improves a lightweight model based on the YOLO framework and names it LCGSC-YOLO. The LCNet is employed to reconstruct the backbone network, which significantly decreases the complexity of the model. The GSConv module and the VOVGSCSP module are adopted into the neck network, which reduces the model parameters and computations while enhancing the feature fusion capability. The CA attention mechanism embedded in the network effectively alleviates the problem of degradation of detection accuracy caused by model lightweighting. Through experimental analysis and comparison, the mAP of LCGSC-YOLO is 95.5% and the inference speed is 53 FPS, which satisfies the requirements of practical applications. Therefore, this method can provide technical support for lightweight deployment of embedded devices in apple leaf disease detection.

However, this study may have some limitations in terms of data diversity and changes in environmental conditions. Firstly, the diversity of plant varieties, growth stages, and pests and diseases in agricultural scenarios requires a broadly representative and comprehensive datasets, but the current datasets only cover several of the most common disease categories, which cannot comprehensively cover all diseases and scenario types. Secondly, frequently changing light conditions can affect the accuracy of the model in predicting diseases. To overcome these limitations, this study plans to extend data collection, increase data diversity, and employ data enhancement techniques and light invariant feature extraction methods to improve the robustness and adaptability of the model.

This research realizes the efficient detection of apple leaf diseases. The main influencing factors, such as environmental conditions and disease categories, were considered during the study to ensure the generalization ability and applicability of the model. However, apple health is also affected by a variety of other factors that need to be further explored in future research. Future research directions include, but are not limited to, the following: firstly, the structure and parameters of the model will be further optimized to improve its applicability and robustness in complex situations. Secondly, this study will pay special attention to various changing situations in natural scenarios and continuously expand the datasets in order to evaluate the performance of the model more comprehensively. In addition, other advanced techniques and methods such as transfer learning, and federated learning will be explored. With these improvements, this study expects to provide more comprehensive and reliable technical support for apple disease detection and management.

## Data Availability

Publicly available datasets were analyzed in this study. This data can be found here: https://drive.google.com/drive/folders/1MRfK5eOm5-6KZTngPzpzjp9gx1NyEvZY?usp=drive_link.

## References

[B1] Ahmed K. Shahidi T. R. Alam S. M. I. Momen S. (2019). “Rice leaf disease detection using machine learning techniques,” in 2019 International Conference on Sustainable Technologies for Industry 4.0 (STI), (Dhaka, Bangladesh: IEEE), 01–05.

[B2] Arsenovic M. Karanovic M. Sladojevic S. Anderla A. Stefanovic D. (2019). Solving current limitations of deep learning based approaches for plant disease detection. Symmetry 11, 01–18. doi: 10.3390/sym11070939

[B3] Attri I. Awasthi L. K. Sharma T. P. Rathee P. (2023). A review of deep learning techniques used in agriculture. Ecol. Inf. 77, 01–22. doi: 10.1016/j.ecoinf.2023.102217

[B4] Barman U. Choudhury R. D. Sahu D. Barman G. G. (2020). Comparison of convolution neural networks for smartphone image based real time classification of citrus leaf disease. Comput. Electron. Agric. 177, 01–09. doi: 10.1016/j.compag.2020.105661

[B5] Bhuiyan M. A. B. Abdullah H. M. Arman S. E. Rahman S. S. Al Mahmud K. (2023). Bananasqueezenet: A very fast, lightweight convolutional neural network for the diagnosis of three prominent banana leaf diseases. Smart Agric. Technol. 4, 01–13. doi: 10.1016/j.atech.2023.100214

[B6] Bi C. Wang J. Duan Y. Fu B. Kang J.-R. Shi Y. (2022). Mobilenet based apple leaf diseases identification. Mobile Networks Appl. 27, 1–9. doi: 10.1007/s11036-020-01640-1

[B7] Chollet F. (2017). “Xception: Deep learning with depthwise separable convolutions,” in Proceedings of the IEEE conference on computer vision and pattern recognition, Las Vegas, Nevada. (IEEE), 1251–1258.

[B8] Cui C. Gao T. Wei S. Du Y. Guo R. Dong S.. (2021). Pp-lcnet: A lightweight cpu convolutional neural network. arXiv preprint arXiv:2109.15099 12, 1–9. doi: 10.48550/arXiv.2109.15099

[B9] Dhaka V. S. Meena S. V. Rani G. Sinwar D. Ijaz M. F. Woźniak M. (2021). A survey of deep convolutional neural networks applied for prediction of plant leaf diseases. Sensors 21, 4749. doi: 10.3390/s21144749 34300489 PMC8309553

[B10] Elbasi E. Zaki C. Topcu A. E. Abdelbaki W. Zreikat A. I. Cina E.. (2023). Crop prediction model using machine learning algorithms. Appl. Sci. 13, 9288. doi: 10.3390/app13169288

[B11] Harakannanavar S. S. Rudagi J. M. Puranikmath V. I. Siddiqua A. Pramodhini R. (2022). Plant leaf disease detection using computer vision and machine learning algorithms. Global Transit. Proc. 3, 305–310. doi: 10.1016/j.gltp.2022.03.016

[B12] Hossin M. Sulaiman M. N. (2015). A review on evaluation metrics for data classification evaluations. Int. J. Data Min. knowledge Manage. process 5, 01–11. doi: 10.5121/ijdkp.2015.5201

[B13] Howard A. Sandler M. Chu G. Chen L.-C. Chen B. Tan M.. (2019). “Searching for mobilenetv3,” in Proceedings of the IEEE/CVF international conference on computer vision, Long Beach Convention & Entertainment Center. (Los Angeles CA, United States: IEEE), 1314–1324.

[B14] Hu L.-Y. Hong Y. A. Zhang J.-Y. Yang-Tian Su L. Gong X.-Q. Kun Z.. (2022). Overexpression of mdmips1 enhances drought tolerance and water-use efficiency in apple. J. Integr. Agric. 21, 1968–1981. doi: 10.1016/S2095-3119(21)63822-4

[B15] Hu Y. Liu G. Chen Z. Liu J. Guo J. (2023). Lightweight one-stage maize leaf disease detection model with knowledge distillation. Agriculture 13, 01–22. doi: 10.3390/agriculture13091664

[B16] Hu J. Shen L. Sun G. (2018). “Squeeze-and-excitation networks,” in Proceedings of the IEEE conference on computer vision and pattern recognition. (Seoul, South Korea: IEEE), 7132–7141.

[B17] Hyson D. A. (2011). A comprehensive review of apples and apple components and their relationship to human health. Adv. Nutr. 2, 408–420. doi: 10.3945/an.111.000513 22332082 PMC3183591

[B18] Jackulin C. Murugavalli S. (2022). A comprehensive review on detection of plant disease using machine learning and deep learning approaches. Measure.: Sensors 24, 01–10. doi: 10.1016/j.measen.2022.100441

[B19] Jiang P. Chen Y. Liu B. He D. Liang C. (2019). Real-time detection of apple leaf diseases using deep learning approach based on improved convolutional neural networks. IEEE Access 7, 59069–59080. doi: 10.1109/ACCESS.2019.2914929

[B20] Jiang F. Lu Y. Chen Y. Cai D. Li G. (2020). Image recognition of four rice leaf diseases based on deep learning and support vector machine. Comput. Electron. Agric. 179, 01–09. doi: 10.1016/j.compag.2020.105824

[B21] Johannes A. Picon A. Alvarez-Gila A. Echazarra J. Rodriguez-Vaamonde S. Navajas A. D.. (2017). Automatic plant disease diagnosis using mobile capture devices, applied on a wheat use case. Comput. Electron. Agric. 138, 200–209. doi: 10.1016/j.compag.2017.04.013

[B22] Justus D. Brennan J. Bonner S. McGough A. S. (2018). “Predicting the computational cost of deep learning models,” in 2018 IEEE international conference on big data (Big Data). (Seattle, WA, USA: IEEE), 3873–3882.

[B23] Kaur P. Harnal S. Gautam V. Singh M. P. Singh S. P. (2022). An approach for characterization of infected area in tomato leaf disease based on deep learning and object detection technique. Eng. Appl. Artif. Intell. 115, 01–12. doi: 10.1016/j.engappai.2022.105210

[B24] Khan A. I. Quadri S. Banday S. Shah J. L. (2022). Deep diagnosis: A real-time apple leaf disease detection system based on deep learning. Comput. Electron. Agric. 198, 107093. doi: 10.1016/j.compag.2022.107093

[B25] Kiani Galoogahi H. Fagg A. Huang C. Ramanan D. Lucey S. (2017). “Need for speed: A benchmark for higher frame rate object tracking,” in Proceedings of the IEEE International Conference on Computer Vision. (Seattle, WA, USA: IEEE), 1125–1134.

[B26] Li S. Li K. Qiao Y. Zhang L. (2022b). A multi-scale cucumber disease detection method in natural scenes based on yolov5. Comput. Electron. Agric. 202, 01–12. doi: 10.1016/j.compag.2022.107363

[B27] Li H. Li J. Wei H. Liu Z. Zhan Z. Ren Q. (2022a). Slim-neck by gsconv: A better design paradigm of detector architectures for autonomous vehicles. arXiv preprint arXiv:2206.02424 120, 01–17. doi: 10.1007/s11554-024-01436-6

[B28] Li H. Shi L. Fang S. Yin F. (2023). Real-time detection of apple leaf diseases in natural scenes based on yolov5. Agriculture 13, 878. doi: 10.3390/agriculture13040878

[B29] Ma N. Zhang X. Zheng H.-T. Sun J. (2018). “Shufflenet v2: Practical guidelines for efficient cnn architecture design,” in Proceedings of the European conference on computer vision (ECCV). (Anchorage, Alaska, United States: Springer), 116–131.

[B30] Maddikunta P. K. R. Hakak S. Alazab M. Bhattacharya S. Gadekallu T. R. Khan W. Z.. (2021). Unmanned aerial vehicles in smart agriculture: Applications, requirements, and challenges. IEEE Sensors J. 21, 17608–17619. doi: 10.1109/JSEN.2021.3049471

[B31] Ngugi L. C. Abelwahab M. Abo-Zahhad M. (2021). Recent advances in image processing techniques for automated leaf pest and disease recognition–a review. Inf. Process. Agric. 8, 27–51. doi: 10.1016/j.inpa.2020.04.004

[B32] Niu Z. Zhong G. Yu H. (2021). A review on the attention mechanism of deep learning. Neurocomputing 452, 48–62. doi: 10.1016/j.neucom.2021.03.091

[B33] Orchi H. Sadik M. Khaldoun M. (2021). On using artificial intelligence and the internet of things for crop disease detection: A contemporary survey. Agriculture 12, 9. doi: 10.3390/agriculture12010009

[B34] Rastogi A. Arora R. Sharma S. (2015). “Leaf disease detection and grading using computer vision technology & fuzzy logic,” in 2015 2nd international conference on signal processing and integrated networks (SPIN). (Noida, India: IEEE), 500–505.

[B35] Redmon J. Divvala S. Girshick R. Farhadi A. (2016). “You only look once: Unified, realtime object detection,” in Proceedings of the IEEE conference on computer vision and pattern recognition. (Long Beach, USA: IEEE), 779–788.

[B36] Redmon J. Farhadi A. (2017). “Yolo9000: better, faster, stronger,” in Proceedings of the IEEE conference on computer vision and pattern recognition. (Salt Lake City,USA: IEEE), 7263–7271.

[B37] Ren S. He K. Girshick R. Sun J. (2015). Faster r-cnn: Towards real-time object detection with region proposal networks. Adv. Neural Inf. Process. Syst. 39, 1137–1149. doi: 10.1109/TPAMI.2016.2577031 27295650

[B38] Roy A. M. Bhaduri J. (2021). A deep learning enabled multi-class plant disease detection model based on computer vision. Ai 2, 413–428. doi: 10.3390/ai2030026

[B39] Sujatha R. Chatterjee J. M. Jhanjhi N. Brohi S. N. (2021). Performance of deep learning vs machine learning in plant leaf disease detection. Microprocessors Microsys. 80, 103615. doi: 10.1016/j.micpro.2020.103615

[B40] Sun T. Xing H. Cao S. Zhang Y. Fan S. Liu P. (2022). A novel detection method for hot spots of photovoltaic (pv) panels using improved anchors and prediction heads of yolov5 network. Energy Rep. 8, 1219–1229. doi: 10.1016/j.egyr.2022.08.130

[B41] Tian H. Wang T. Liu Y. Qiao X. Li Y. (2020). Computer vision technology in agricultural automation—a review. Inf. Process. Agric. 7, 1–19. doi: 10.1016/j.inpa.2019.09.006

[B42] Wang Y. Wang Y. Zhao J. (2022). Mga-yolo: A lightweight one-stage network for apple leaf disease detection. Front. Plant Sci. 13. doi: 10.3389/fpls.2022.927424 PMC9441945 36072327

[B43] Xu W. Wang R. (2023). Alad-yolo: An lightweight and accurate detector for apple leaves. Front. Plant Sci. 14. doi: 10.3389/fpls.2023.1204569 PMC10472937 37662152

[B44] Zeng W. Li M. (2020). Crop leaf disease recognition based on self-attention convolutional neural network. Comput. Electron. Agric. 172, 01–13. doi: 10.1016/j.compag.2020.105341

[B45] Zhang J. Meng Y. Yu X. Bi H. Chen Z. Li H.. (2023). Mbab-yolo: A modified lightweight architecture for real-time small target detection. IEEE Access 11, 01–09. doi: 10.1109/ACCESS.2023.3286031

[B46] Zhu S. Ma W. Wang J. Yang M. Wang Y. Wang C. (2023). Eadd-yolo: An efficient and accurate disease detector for apple leaf using improved lightweight yolov5. Front. Plant Sci. 14. doi: 10.3389/fpls.2023.1120724 PMC9996066 36909428

